# Temporal Associations between Weather and Headache: Analysis by Empirical Mode Decomposition

**DOI:** 10.1371/journal.pone.0014612

**Published:** 2011-01-31

**Authors:** Albert C. Yang, Jong-Ling Fuh, Norden E. Huang, Ben-Chang Shia, Chung-Kang Peng, Shuu-Jiun Wang

**Affiliations:** 1 Department of Psychiatry, Chu-Tung Veterans Hospital, Hsin-Chu County, Taiwan; 2 Institute of Clinical Medicine, National Yang-Ming University, Taipei, Taiwan; 3 Center for Dynamical Biomarkers and Translational Medicine, National Central University, Chungli, Taiwan; 4 Department of Neurology, Neurological Institute, Taipei Veterans General Hospital, Taipei, Taiwan; 5 Department of Neurology, National Yang-Ming University School of Medicine, Taipei, Taiwan; 6 Research Center for Adaptive Data Analysis, National Central University, Chungli, Taiwan; 7 Department of Statistics and Information Science, Fu Jen Catholic University, Taipei County, Taiwan; 8 Margret and H. A. Rey Institute for Nonlinear Dynamics in Medicine, Beth Israel Deaconess Medical Center/Harvard Medical School, Boston, Massachusetts, United States of America; University of Liverpool, United Kingdom

## Abstract

**Background:**

Patients frequently report that weather changes trigger headache or worsen existing headache symptoms. Recently, the method of empirical mode decomposition (EMD) has been used to delineate temporal relationships in certain diseases, and we applied this technique to identify intrinsic weather components associated with headache incidence data derived from a large-scale epidemiological survey of headache in the Greater Taipei area.

**Methodology/Principal Findings:**

The study sample consisted of 52 randomly selected headache patients. The weather time-series parameters were detrended by the EMD method into a set of embedded oscillatory components, i.e. intrinsic mode functions (IMFs). Multiple linear regression models with forward stepwise methods were used to analyze the temporal associations between weather and headaches. We found no associations between the raw time series of weather variables and headache incidence. For decomposed intrinsic weather IMFs, temperature, sunshine duration, humidity, pressure, and maximal wind speed were associated with headache incidence during the cold period, whereas only maximal wind speed was associated during the warm period. In analyses examining all significant weather variables, IMFs derived from temperature and sunshine duration data accounted for up to 33.3% of the variance in headache incidence during the cold period. The association of headache incidence and weather IMFs in the cold period coincided with the cold fronts.

**Conclusions/Significance:**

Using EMD analysis, we found a significant association between headache and intrinsic weather components, which was not detected by direct comparisons of raw weather data. Contributing weather parameters may vary in different geographic regions and different seasons.

## Introduction

Headache is one of the most challenging conditions confronting clinicians in their daily practice [1]. Headache sufferers frequently describe weather changes as triggers for headache onset or the worsening of ongoing headache symptoms. Although many people in the general population believe that there is an association between headache and weather [2], early studies examining this possibility have yielded inconsistent results [3], [4], [5], [6]. The variability in prior findings may be due, at least in part, to the lack of systemic comparisons of a wide range of climatic parameters in relation to headache [7], as well as the lack of adequate analytical methods to investigate weather data, which are often highly dynamic on multiple time scales.

Recent reports have indicated that several weather parameters may be associated with headache, including ambient temperature, barometric pressure, relative humidity, and wind speed [7], [8], [9], [10], [11]. However, few studies have examined the temporal relationship between weather and headache. A broad investigation of the temporal effects of weather change on headache attacks is not only essential for identifying causal links between headache and headache triggers, but would also allow clinicians to more effectively manage their headache patients.

Weather patterns reflect a complex interaction among multiple meteorological factors. As a result, consecutive weather time series often show complex fluctuations over time, and their association with headache incidence is difficult to analyze by conventional methods. In the present study, we applied an adaptive-based method of empirical mode decomposition (EMD) [12], [13] to detrend weather data. The EMD method provides a generic algorithm to decompose a complex time series [13] into a set of intrinsic oscillations, called intrinsic mode functions (IMFs), which are orthogonal to one another and can therefore be treated as independent factors, making this method suitable for the challenge of analyzing the temporal association between weather and headache.

We examined the headache diary data from an epidemiological study of migraine conducted in 1997 in the Greater Taipei area [14]. Using multiple linear regression analysis, we aimed to evaluate the association of decomposed weather IMFs with headache incidence and to analyze separately the temporal relationship between headache and decomposed weather variables during warm and cold periods.

## Materials and Methods

### Subjects

The Greater Taipei Migraine Study was a population-based survey using a validated questionnaire that was conducted from August 1997 to June 1998 [14]. The target population comprised all individuals (age≥15 years) in 1400 randomly selected households. Migraine diagnoses were made according to the classification criteria of migraine without aura proposed by the first version of the International Classification of Headache Disorders (ICHD-1), except that attacks with a duration of between 2 hours and 4 hours were also included. Trained interviewers administered the questionnaire interview to each participant in person. Of the 4434 eligible subjects in the 1211 respondent households, 3377 (76%) completed the questionnaire. The 1-year prevalence of migraine was 9.1% (female/male: 14.4%/4.5%) [14].

Among those subjects reporting headaches more than 2 days per month, 52 subjects (94.2% female; mean age 28.6±10.4 years; range 15–48 years) randomly sampled from the community kept headache diaries from August 7 to December 31, 1997 (147 days). The headache diary was designed according to the ICHD-1 to include the information regarding the diagnoses of migraine or tension-type headache, such as headache duration, aura, location, character and intensity of pain and accompanying symptoms: nausea, vomiting, photophobia and phonophobia [15]. Each participant reported headache onset dates and rated each headache's intensity as mild, moderate or severe. Daily headache incidence was calculated as the proportion of subjects reporting headache out of the total number of subjects. The Institutional Review Board of Taipei Veterans General Hospital approved examination of the headache diary data derived from this epidemiological study.

### Weather data

Local weather data measured at corresponding times in the central Taipei area was provided by the Central Weather Bureau, Taiwan. The greater Taipei area is in a subtropical region characterized by a cool winter and a hot summer. The average annual temperature is about 22°C, ranging from 15°C in winter to 27°C in summer. Large temperature fluctuations, accompanied by dramatic changes in pressure, humidity, or sunshine duration, are often seen in winter when cold fronts periodically pass through the area [16].

Primary weather variables included ambient temperature, barometric pressure, relative humidity, maximum wind speed, and total hours of sunshine on each day. All weather variables were average daily values. Since a weather change, rather than an absolute value, could also serve as a headache trigger, a gradient change of each weather time series was also derived, defined as the difference in the average of a weather variable from the previous day.

### EMD

The EMD method was developed to detrend and identify intrinsic oscillations embedded in a complex signal [12] and has been widely applied in multiple disciplines [17], [18], [19]. Unlike Fourier-based time series analysis, EMD holds no *a priori* assumption for underlying structures of the time series and is therefore suitable for analyzing time series consisting of multiple periodic components (e.g., climatic data or biomedical signals). The decomposition is based on the simple assumption that any data consist of a finite number of intrinsic components of oscillations. Each component of oscillation, termed an IMF, is sequentially decomposed from the original time series by a sifting process.

Briefly, the sifting process is comprised of the following steps: 1) connecting local maxima or minima of a targeted signal to form the upper and lower envelopes by natural cubic spline lines, respectively; 2) extracting the first prototype IMF by estimating the difference between the targeted signal and the mean of the upper and lower envelopes; and 3) repeating the above procedures to produce a set of IMFs represented by a certain frequency-amplitude modulation at a characteristic time scale. The decomposition process is completed when no more IMFs can be extracted, and the residual component (not IMF) is treated as the overall trend of the raw data. Although IMFs are empirically determined, they remain orthogonal to one another and may therefore contain independent physical meaning that is relevant to other parameters [13], [20].

To overcome the problem of scale mixing, a new noise-assisted method was employed to improve EMD: the ensemble EMD [21], [22]. This method defines the true IMF components as the average of an ensemble of trials (N = 10,000 in our study), each consisting of the signal plus white noise of finite amplitude. The standard deviation (SD) of added white noise is 0.3 of the SD in the original time series. The added noise in each trial is cancelled out in the ensemble mean of large trials. Of note, the uniformly added white noise helps to project the decomposition of IMFs onto comparable scales independent of the nature of original signals, thus reducing the problem of scale mixing. Although the noise introduced by the ensemble EMD algorithm may induce distortions to IMFs, the degree of distortion can be reduced by using a large number of trials. The degree of distortion, calculated as 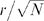 (where r is the amplitude ratio of noise to signal), was 0.003 in our study.

The EMD method served two purposes in this study: (1) to decompose different weather time series into IMFs; each oscillated at certain time scales, and (2) to de-trend (i.e., to remove residual component from raw data) the time series of weather data to produce a zero-mean distribution, thus reducing spurious regression and multi-collinearity in subsequent multiple linear regression analyses.

A publicly available EMD algorithm based on Matlab software (version 2007; The Mathworks, Natick, Massachusetts) was used in this study (http://rcada.ncu.edu.tw/research1.htm).

### Statistical analysis

SPSS for Windows Version 15.0 (SPSS Inc., Chicago, IL) was used for statistical analyses. Skewness was used to evaluate the asymmetry of the distribution of headache incidence. Pearson correlations were used to evaluate the relations among daily headache incidence and raw weather time series data. The relationship between daily headache incidence (dependent variable) and decomposed weather IMFs (independent variables) was studied using linear regression analyses with forward stepwise methods, which were performed separately for models involving single or multiple weather variables. The forward stepwise method was chosen to avoid colinearity because the weather variables were highly correlated. The variance inflation factor (VIF) was estimated for all weather IMFs and a VIF value of 10 or greater is considered to be an indication of significant collinearity. In the model involving single weather variables, decomposed IMFs from both original time series and gradient changes of time series were entered into the regression analyses. All weather IMFs identified in single weather variable models were then selected in the final model of regression analysis (i.e., multiple weather variables model) to compute the most significant predictors of headache incidence. In each model, the total variance (R^2^) in headache incidence explained by the identified IMFs was determined. We analyzed the data from the entire study period, as well as from the first and second half of study period separately, as the first half was from August 7 to October 18, during the warm period, whereas the second half was from October 19 to December 31, during the cold period, which included six documented cold fronts [16]. A two-tailed p value of less than 0.05 was required for statistical significance in all analyses.

## Results

### Participants and headache incidence data

Based on the ICHD-1, of the 52 participants, 19 (37%) were diagnosed as having migraines, 12 (23%) with migrainous disorder, and 21 (40%) with tension-type headaches [23]. No subjects had a medical history of diabetes, and only two (3.8%) had been diagnosed with hypertension. Of note, four subjects (7.7%) drank coffee daily, one (1.9%) drank caffeinated sodas daily, and two (3.8%) drank caffeinated tea daily.

In total, 1809 diary entries documenting 195 headache attacks (119 mild intensity, 59 moderate, and 17 severe) were recorded. The average length of the headache diary per subject was 39±29 days (range: 12–141 days). On average, approximately 12 subjects per day had a diary record of headache during the study period. Mean daily headache incidence was 10.9% (range: 0–41.6%). Evaluation of normal distribution in headache incidence data indicated a skewness of 0.95±0.20.

### Weather time series

**Figure 1:**
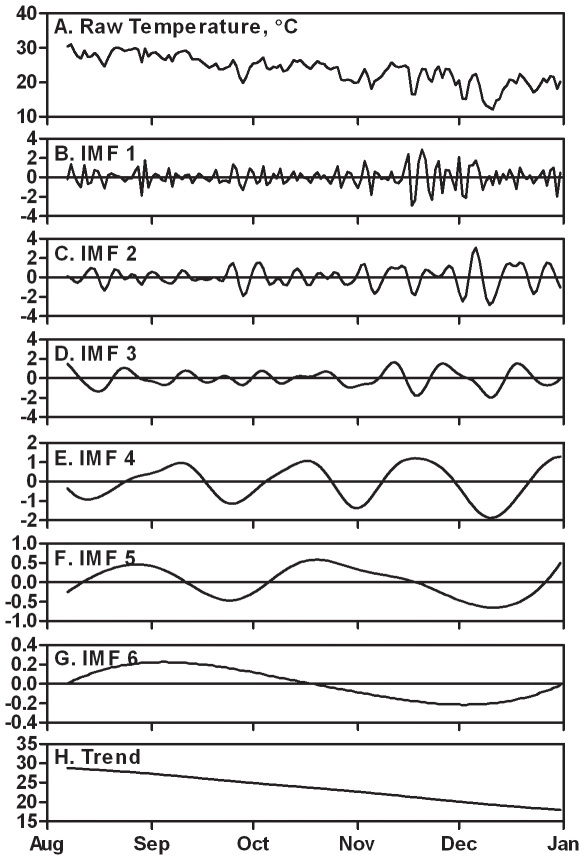
Decomposition of temperature time series. A) Ambient temperature from August 7 to Dec 31, 1997. B-G) IMFs decomposed from raw temperature time series using empirical mode decomposition analysis. H) Overall trend. The raw temperature data can be recovered by summing all IMFs (panels B-G) and the overall trend (H). Only decomposed IMFs (excluding trend component) were entered into the regression models.

Table 1 summarizes the Pearson correlations between daily headache incidence and raw weather time series. Temperature showed a significant correlation with pressure (r = −0.77, p<0.001), sunshine duration (r = 0.49, p<0.001), and relative humidity (r = −0.52, p<0.001). However, no significant correlations were found between headache incidence and the raw data of any meteorological factor. Figure 1 shows the results of decomposition of weather time series by the EMD method, using temperature as an example. The decomposition of raw temperature data (Figure 1A) yielded a total of six IMFs (Figure 1B–1G) and a residual (overall trend; Figure 1H).

**Table 1:** Correlation coefficients among raw headache incidence and weather variables.

	Headache incidence	Temperature	Pressure	Humidity	Sunshine duration	Maximal wind speed
Headache incidence	1.00	−0.02	0.03	−0.12	0.07	−0.10
Temperature		1.00	−0.78**	−0.23**	0.49**	−0.11
Pressure			1.00	−0.08	−0.17*	−0.01
Humidity				1.00	−0.52**	−0.15
Sunshine duration					1.00	−0.07
Maximal wind speed						1.00

*p<0.05 level (2-tailed),

**p<0.01 level (2-tailed).

### Prediction of daily headache incidence by single weather variables

We first examined the association of headache incidence with each weather parameter, using headache incidence as the dependent variable and decomposed weather IMFs as predictors, as shown in Table 2 (see Table S1 for the detailed list of IMFs). For the headache incidence data from the entire study period, the greatest amounts of variance were accounted for by sunshine duration (R^2^ = 0.166, p<0.001), followed by temperature (R^2^ = 0.108, p = 0.001), humidity (R^2^ = 0.049, p = 0.007), maximal wind speed (R^2^ = 0.035, p = 0.022) and pressure (R^2^ = 0.030, p = 0.036).

**Table 2:** Models of headache incidence and single weather variables.

Weather variable	Model summary				
	R	R^2^	SE	F	*P*
**Entire period**					
Temperature	0.329	0.108	8.375	5.784	0.001
Barometric pressure	0.173	0.030	8.674	4.473	0.036
Relative humidity	0.222	0.049	8.588	7.496	0.007
Sunshine duration	0.420	0.166	8.127	7.065	<0.001
Maximal wind speed	0.188	0.035	8.649	0.873	0.022
**Warm period (Aug 7 to Oct 18)**					
Maximal wind speed	0.248	0.062	7.640	4.655	0.034
**Cold period (Oct 19 to Dec 31)**					
Temperature	0.444	0.197	8.632	8.723	<0.001
Barometric pressure	0.313	0.098	9.085	7.842	0.007
Relative humidity	0.420	0.177	8.742	7.615	0.001
Sunshine duration	0.448	0.201	8.612	8.922	<0.001

Headache incidence data from the warm and cold periods were also analyzed. For the warm period, a significant model was derived for maximal wind speed (R^2^ = 0.062, p = 0.034), but not for temperature, pressure, humidity, or sunshine duration. During the cold period, the highest variance was accounted for by temperature (R^2^ = 0.197, p<0.001) and sunshine duration (R^2^ = 0.201, p<0.001), followed by humidity (R^2^ = 0.177, p = 0.001), and pressure (R^2^ = 0.098, p = 0.007). However, no significant model was derived during the cold period for maximal wind speed. There was no significant effect of lead or lag scale (±1–5 days) on regression models (data not shown).

### Prediction of daily headache incidence by multiple weather variables

In the multiple weather variables model, all significant variables found in the single weather variable model were used in a forward stepwise multiple linear regression analysis, as shown in Table 3. The regression model showed that higher associations were found between daily headache incidence and weather IMFs in the cold period (R^2^ = 0.33, p<0.001) than in the warm period (R^2^ = 0.06, p = 0.034). In the warm period, maximal wind speed was the only predictor of headache incidence (3^rd^ IMF: r = −0.248, p = 0.034). In the cold period, headache incidence was predicted by a set of IMFs related to temperature (1^st^ IMF: r = 0.315, p = 0.008; 4^th^ IMF of gradient change: r = −0.282, p = 0.017) and sunshine duration (2^nd^ IMF: r = 0.352, p = 0.003; 4^th^ IMF of gradient change: r = 0.406, p<0.001). These temporal correlations in the cold period occurred at two distinct time scales with frequencies of 3.4 to 7.6 days per cycle and cycles lasting 34.4 to 41.4 days.

**Table 3:** Final model of headache incidence and multiple weather variables.

Weather IMFs	Model Summary					
	IMF order	Average period (days)	*β*	SE	Partial correlation	*P*
**Warm period (Aug 7 to Oct 18)**: R^2^ = 0.06, F = 4.655, p = 0.034						
Max. wind speed	3^rd^	12.2	−1.129	0.523	−0.248	0.034
**Cold period (Oct 19 to Dec 31)**: R^2^ = 0.33, F = 8.594, p<0.001						
Temperature	1^st^	3.4	2.375	0.863	0.315	0.008
Sunshine duration (gradient change)	2^nd^	8.9	1.906	0.610	0.352	0.003
Temperature (gradient change)	4^th^	41.4	−7.047	2.889	−0.282	0.017
Sunshine duration	4^th^	34.4	3.053	0.826	0.406	<0.001

Note: Only weather IMFs identified in Table 2 were used in this model.

**Figure 2:**
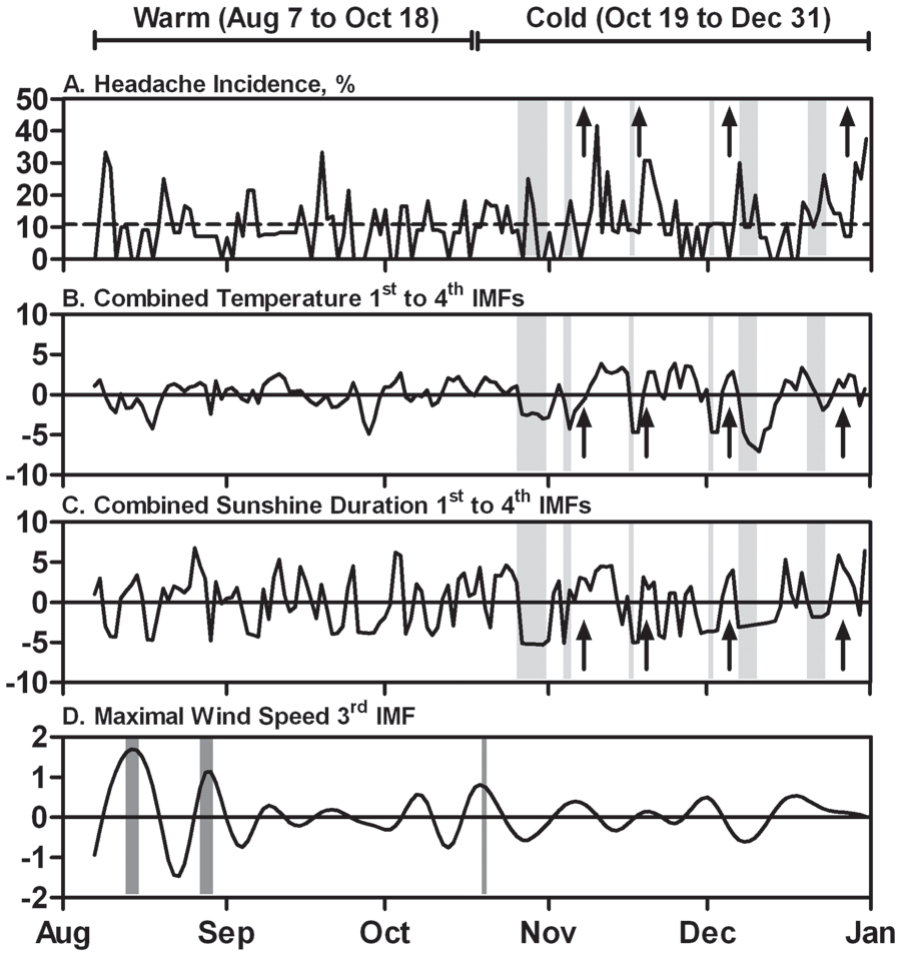
Comparison of headache incidence and weather IMFs. A) Headache incidence data. The dashed line indicates the average headache incidence across the entire study period. B) 1^st^ to 4^th^ temperature IMFs combined. C) 1^st^ to 4^th^ sunshine IMFs combined. Dark grey shaded areas indicate the documented typhoons in the warm period, whereas light grey shaded areas indicate documented cold fronts in the cold period [16]. Arrows indicate the aftermath of cold fronts that coincided with increased headache incidence.

To illustrate the temporal impact of weather on headache incidence, Figure 2 shows the headache incidence, 3^rd^ maximal wind speed IMF, as well as temperature and sunshine data combined from the 1^st^ and 4^th^ IMFs, respectively. During the warm period, increased headache incidence coincided with the increased amplitude of maximal wind speed, which was compatible with two out of three documented typhoons. During the cold period, the aftermath of four out of six cold fronts coincided with increased headache incidence.

## Discussion

Using EMD analysis to decompose weather time series into identifiable oscillations, we found a significant association between headache incidence and intrinsic weather patterns, a relationship which was not detected in our analysis of the raw weather data. While many meteorological factors were strongly associated with headache incidence during the cold period, these associations were weak or absent during the warm period. Among pooled weather variables, IMFs derived from temperature and sunshine duration data accounted for up to 33.3% of the variance in headache incidence during the cold period. Our results therefore support the notion that changes in weather are associated with headache occurrence, particularly in the cold season.

In our study, the temporal association between headache and most meteorological variables during the cold period appeared to coincide with cold fronts. A cold front often results in considerable drops in temperature and sunshine duration, as well as increases in barometric pressure and relative humidity, and these weather changes recover gradually after the resolution of the cold front [24]. Increased headache incidence was associated with increased temperature and sunshine duration, which is temporally correlated to the aftermath of most documented cold fronts. While the mechanism of impact of weather on headache remains largely unknown, headache is known to be associated with hemodynamic changes, which might be aggravated by the cold weather. Our findings may imply that cold fronts either play a role in precipitating headache attacks or have a priming effect on headache occurrence. Of interest, we found a weak correlation between headache incidence and maximal wind speed data exclusively during the warm period. Whether this correlation is due to an influence of typhoons may warrant further investigation.

Our study provides new insights into the association of headache with weather in several respects. First, the subjects recruited to participate in our study were from an epidemiological study [14], and the headache diaries were originally used for other purposes. Therefore, selection bias due to either subjective sensitivity to weather conditions or hospital-based studies was minimized in this sample. Second, this study is the first to demonstrate that EMD can effectively decompose weather time series into IMFs with identifiable oscillations, allowing investigation of the temporal relationship between headache incidence and changes in weather variables. Prior studies have also found that the empirically decomposed IMFs reflect certain physical meanings related to underlying mechanisms. For example, EMD has been used successfully to isolate travelling waves in dengue hemorrhagic fever incidence across Thailand [17] and to evaluate the risk of stroke by identifying oscillations in cerebral blood flow related to cerebral auto-regulation [19].

The inconsistency of findings from previous studies examining the putative relationship between headache attacks and weather parameters may have been due to the variability of weather triggers in different geographic locations. In the Boston area of the USA, a self-report survey found that low barometric pressure could trigger headaches [7], whereas a hospital-based study from the same area observed that a transient increase in emergency visits for migraine was associated with higher ambient temperature [10]. However, another study conducted in Ottawa, Canada showed no association between emergency visits for migraine and weather conditions [25]. Studies conducted in Italy and Germany support an association between headache incidence and temperature [8], [26]. In the Alberta region of Canada, Chinook winds (warm winds with high speed) have been linked to headache attacks [9], [11]. Of note, none of these studies examined the temporal associations between weather parameters and headache incidences, as in our study. In addition, most of these studies were conducted in temperate or high-latitude areas, which are quite different from the subtropical climate patterns in Taiwan. It is possible that weather patterns in distinct geographic locations may differentially affect headache attacks.

This study has some limitations, including the length of the headache diaries and the underrepresentation of males. The distribution of headache incidence was mildly skewed (skewness = 0.95) and may limit the interpretation of results from linear regression analysis. In addition, because of the limited number of participants, this study only reported total headache incidence rather than the specific incidences of particular headache types, such as migraine or tension-type headache, which might have differential sensitivity to weather changes. Weather variables are continuous exposure measures and may not be related to headache incidence data in a linear fashion. We tested the assumption by applying generalized additive models and found that the model results were unstable, partly due to collinearity in the weather data. For this reason, even linear relationship may overly simplify the association between headache and weather data, conventional linear regression analysis with forward stepwise method may appear more suitable when the data shows high collinearity.

Notwithstanding the aforementioned limitations, we propose that the methodology used in this study can serve as a general framework for investigating the temporal association between headache and weather variables in other climatic regions. The impact of weather on headache can thereby be comprehensively delineated by integrating reports from locations throughout the world. The impact of certain trigger factors may outweigh others in certain populations. For example, one study showed that menstruation-related migraine did not exhibit seasonal patterns [27]. A prospective study with a larger population and a longer time period (e.g., ≥1 year) is warranted to confirm these findings. Such a study may also help to reveal the complex interactions between headache, weather change, and other trigger factors.

## Supporting Information

Table S1 Headache incidence and single weather variables.
(0.10 MB DOC)
