# Supplementary material for: Temporal Associations between Weather and Headache: Analysis by Empirical Mode Decomposition
Source: PLoS One. 2011 Jan 31;6(1):e14612. doi: 10.1371/journal.pone.0014612 (PMC3031498; doi:10.1371/journal.pone.0014612)
Supplement: Table S1 — Headache incidence and single weather variables. (0.10 MB DOC) [file pone.0014612.s001.doc]

**Table S1.** Headache incidence and single weather variables.

| **Weather IMFs** | **Model Summary** | | | | |
| --- | --- | --- | --- | --- | --- |
|  | **Average period (days)** | ***β*** | **SE** | **Partial correlation** | ***P*** |
| **Temperature – entire period** (R^2^ = 0.108, F = 5.784, p = 0.001) | | | | | |
| 1^st^ | 3.4 | 1.469 | 0.734 | 0.165 | 0.047 |
| 4^th^ | 41.4 | 1.985 | 0.796 | 0.204 | 0.014 |
| 4^th^ gradient change | 21.7 | -6.155 | 2.724 | -.186 | 0.025 |
|  |  |  |  |  |  |
| **Temperature – warm period:** no significant model was derived | | | | | |
|  | | | | | |
| **Temperature – cold period** (R^2^ = 0.197, F = 8.723, p < 0.001) | | | | | |
| 4^th^ | 41.4 | 3.198 | 0.993 | 0.357 | 0.002 |
| 4^th^ gradient change | 21.7 | -6.704 | 3.145 | -0.245 | 0.037 |
|  |  |  |  |  |  |
| **Pressure – entire period** (R^2^ = 0.030, F = 4.473, p = 0.036) | | | | | |
| 4^th^ | 41.9 | -0.889 | 0.420 | -0.173 | 0.036 |
|  |  |  |  |  |  |
| **Pressure – warm period:** no significant model was derived | | | | | |
|  | | | | | |
| **Pressure – cold period** (R^2^ = 0.098, F = 7.842, p = 0.007) | | | | | |
| 4^th^ | 41.9 | -1.547 | 0.553 | -0.313 | 0.007 |
|  |  |  |  |  |  |
| **Humidity – entire period** (R^2^ = 0.049, F = 7.496, p = 0.007) | | | | | |
| 4^th^ | 36.7 | -0.420 | 0.154 | -0.222 | 0.007 |
|  |  |  |  |  |  |
| **Humidity – warm period:** no significant model was derived | | | | |  |
|  | | | | | |
| **Humidity – cold period** (R^2^ = 0.177, F = 7.615, p = 0.001) | | | | | |
| 4^th^ | 36.7 | -1.279 | 0.350 | -0.398 | <0.001 |
| 5^th^ gradient change | 58.5 | 5.886 | 2.603 | 0.259 | 0.027 |
|  |  |  |  |  |  |
| **Sunshine duration – entire period** (R^2^ = 0.166, F = 7.065, p < 0.001) | | | | | |
| 2^nd^ | 8.9 | -1.046 | 0.402 | -0.213 | 0.010 |
| 3^rd^ | 16.8 | 1.958 | 0.600 | 0.264 | 0.001 |
| 5^th^ gradient change | 42.9 | 13.292 | 3.724 | 0.287 | <0.001 |
| 6^th^ gradient change | 137.0 | -7.649 | 3.376 | -0.187 | 0.025 |
| **Sunshine duration – warm period:** no significant model was derived | | | | | |
|  | | | | | |
| **Sunshine duration – cold period** (R^2^ = 0.201, F = 8.922, p < 0.001) | | | | | |
| 4^th^ | 34.4 | 3.231 | 0.887 | 0.397 | 0.001 |
| 2^nd^ gradient change | 7.6 | 1.361 | 0.618 | 0.253 | 0.031 |
|  |  |  |  |  |  |
| **Maximal wind Speed – entire period** (R^2^ = 0.035, F = 0.873, p = 0.022) | | | | | |
| 3^rd^ | 12.2 | -1.254 | 0.543 | -0.188 | 0.022 |
|  |  |  |  |  |  |
| **Maximal wind Speed – warm period** (R^2^ = 0.062, F = 4.655, p = 0.034) | | | | | |
| 3^rd^ | 12.2 | -1.129 | 0.523 | -0.248 | 0.034 |
|  |  |  |  |  |  |
| **Maximal wind Speed – cold period:** no significant model was derived | | | | | |
|  | | | | | |
